# Risk factors for high-altitude headache upon acute high-altitude exposure at 3700 m in young Chinese men: a cohort study

**DOI:** 10.1186/1129-2377-14-35

**Published:** 2013-04-11

**Authors:** Shi-Zhu Bian, Ji-Hang Zhang, Xu-Bin Gao, Ming Li, Jie Yu, Xi Liu, Jun-Qing Dong, Guo-Zhu Chen, Lan Huang

**Affiliations:** 1 Institute of Cardiovascular Diseases of PLA; Department of Cardiology, Xinqiao Hospital, Third Military Medical University, Chongqing, 400037, China

**Keywords:** High-altitude headache, Risk factors, Acute, High altitude, Exposure

## Abstract

**Background:**

This prospective and observational study aimed to identify demographic, physiological and psychological risk factors associated with high-altitude headache (HAH) upon acute high-altitude exposure.

**Methods:**

Eight hundred fifty subjects ascended by plane to 3700 m above Chengdu (500 m) over a period of two hours. Structured Case Report Form (CRF) questionnaires were used to record demographic information, physiological examinations, psychological scale, and symptoms including headache and insomnia a week before ascending and within 24 hours after arrival at 3700 m. Binary logistic regression models were used to analyze the risk factors for HAH.

**Results:**

The incidence of HAH was 73.3%. Age (*p* =0.011), physical labor intensity (PLI) (*p* =0.044), primary headache history (*p* <0.001), insomnia (*p* <0.001), arterial oxygen saturation (SaO_2_) (*p* =0.001), heart rate (HR) (*p* =0.002), the Self-Rating Anxiety Scale (SAS) (*p* <0.001), and the Epworth Sleepiness Scale (ESS) (*p* <0.001) were significantly different between HAH and non-HAH groups. Logistic regression models identified primary headache history, insomnia, low SaO_2_, high HR and SAS as independent risk factors for HAH.

**Conclusions:**

Insomnia, primary headache history, low SaO_2_, high HR, and high SAS score are the risk factors for HAH. Our findings will provide novel avenues for the study, prevention and treatment of HAH.

## Background

Headache has been described as the most frequent complaint during acute high-altitude exposure [1-3]. It is also the primary symptom of acute mountain sickness (AMS) in the diagnostic criteria of the Lake Louise Scoring System (LLS). High-altitude headache (HAH) has been defined by the International Headache Society (IHS) as the headache that occurs within 24 hours after ascending to 2500 m or above and subsides within 8 hours after descending [4,5]. HAH refers to a number of pathophysiological processes, some of which are coincident with a primary headache at low altitude [6]. The HAH is also caused by high-altitude environment, but differs from airplane headache which may be caused by atmospheric pressure and is characterized by the sudden onset of a severe head pain exclusively in relation to airplane flights [7]. HAH affects tourists and labor workers due to its high incidence and limitations on daily living and work [8]. The causes, prophylaxis and treatment of HAH are still not comprehensively understood. Thus, it is urgent to identify potential HAH risk factors to design more specific and effective medical prevention and treatment strategies for HAH.

In the past decades, studies on the risk factors of HAH have been emerging. However, the results have been inconsistent. It has been reported that younger age is a risk factor for HAH [9]. However, another study indicated that age, body mass index (BMI), altitude of residence, smoking history, regular caffeine intake and self-estimated level of performance were not significantly different between HAH-positive (HAH+) and non-HAH (HAH-) groups [10]. History of migraine, low arterial oxygen saturation (SaO_2_) value, high rating of perceived exertion and low fluid intake contribute to the development of HAH [10]. However, there are many potential risk factors for HAH which have not yet been indicated such as the rise of blood pressure (BP) upon acute high-altitude exposure, sleeping [11], and the insomnia and its associated distress [12]. Previously, Burtscher et al. [10] performed a valuable investigation at 2200–3817 m with subjects recruited for 3 months to focus on smoking, alcohol consumption, migraine history, heart rate (HR) and SaO_2_.

We propose that demographic and physiological histories, psychological and psychological factors are risk factors for HAH and may cause HAH. To systematically identify more comprehensive risk factors of HAH within unified altitude and duration, we performed a field study that was acute high-altitude exposure (All of the participants were brought to a location at 3700 m by plane within 2 hours). The potential demographic (age, BMI, physical labor intensity [PLI], smoking and alcohol consumption), history (primary headache, athletic training and previous high-altitude exposure), physiological (insomnia, Epworth Sleepiness Scale (ESS), BP, SaO_2_ and HR) and psychological (Self-Rating Anxiety Scale, SAS) risk factors associated with HAH were evaluated in this study.

## Methods

### Participants and procedures

#### Participants

Eight hundred fifty subjects who lived at 500 m and ascended to 3700 m for work participated in the field trials according to the inclusive and exclusive criteria. To participate in the studies, subjects had to be healthy males between the ages of 18 and 45 years old. Subjects with any one of the following conditions were excluded: respiratory diseases, cardiovascular diseases, malignant tumors and liver and kidney dysfunction, and people with psychiatric disorder or neuroses that could not completed the questionnaires. The subjects did not take medication or receive any intervention before ascending.

Participants who agreed to participate in the study were familiar with the purpose and process of this study and signed informed consent forms before the trial. The study was approved by the Ethics Committee of Xinqiao Hospital, the Second Clinic Medical College of Third Military Medical University.

### Procedure

Eight hundred fifty volunteers ascended to 3700 m (Lhasa) in 2 hours by plane from 500 m (Chengdu in Sichuan province). Structured case report form (CRF) questionnaires were used to record demographic data (age, BMI, smoking and alcohol consumption), physiological data (insomnia, ESS, BP, SaO_2_ and HR), medical history [primary headache (subjects’ self-reporting), regular athletic training (>3 month per year) and previous high-altitude exposure (>2500 m) within the past 6 months], psychological scale and symptoms related to AMS (headache, dizziness, insomnia, gastrointestinal symptoms and fatigue/weakness).

The questionnaires of SAS and ESS were shown in Additional file 1 and Additional file 2, and both were translated into Chinese when used in the trial [13,14]. Insomnia assessment: 0 = sleep as usual; 1 = sleep not as good as usual; 2 = difficult to sleep, and 3 = wake up several times at night.

PLI was divided into 3 levels: 1 = office workers, 2 = mild physical labor workers, and 3 = heavy physical labor workers. Headache was represented by 0 to 3 as the severity: 0 = without headache, 1 = mild headache, 2 = moderate headache, and 3 = severe headache. Smoking and alcohol consumption were classified as follows: 0 = no smoking/drinking history, 1 = past smoking/drinking history, and 2 = present smoking/drinking (smoking and drinking regularly). Headache (AMS-headache) and other symptoms in relation with AMS such as dizzy, insomnia, gastrointestinal symptoms and fatigue were included in the CRFs [15]. The HAH relies on the patient-reported headache in the CRFs which have been interpreted by the physicians.

Three physiological signs, BP, SaO_2_ and HR, were measured after resting for 30 min in sitting position.

The BP and HR were examined by sphygmomanometer (HEM-6200, OMRON, China). SaO_2_ was measured by Pulse Oximeter (NONIN-9550, Nonin Onyx, America).

All of the questionnaires and measurements were non-invasive. All of the trial procedures were performed at 500 m within one week before ascending in Chengdu and within 24 h after arrival at 3700 m (Lhasa, around 13:00 pm from June 21st-25th, 2012 and examinations were performed around 8:00–11:00 am in the next morning upon arrival. The minimal and the maximal time from arrival to the examination were 19 hours and 22 hours respectively). The subjects stayed at 3700 m for a week before leaving for the next work place.

### Statistical analysis

The CRFs were rejected if the demographic information, BP, SaO_2_ and HR, were not completed.

Normally distributed variables were expressed as the means ± standard deviations (SD), while the non-normally distributed variables were expressed as medians (inter-quartile ranges). Independent T tests were used to compare two means of normally distributed variables between HAH+ and HAH- groups at 3700 m. Comparisons of the above-mentioned parameters between 500 m and 3700 m altitude were employed by paired-sample T tests. The Mann–Whitney U-test was applied to evaluate differences between ordinal or non-normally distributed data. Differences were considered statistically significant if *p* < 0.05. A variable with *p* < 0.05 was considered a potential risk factor in preliminary screening, and the variable was subjected to an adjusted binary logistic regression model to identify the adjusted independent risk factors for HAH through backward stepwise regression. The statistical analyses were performed using SPSS 19.0 for Windows. Statisticians from Third Military Medical University reviewed all of the statistical methods and results.

## Results

We collected 793 (excluding 34 lost follow-up and 23 uncompleted CRFs) valid CRFs at both 500 m and 3700 m. The mean age of the subjects was 23.03 ± 4.01 years old (mean ± SD), and the mean BMI was 21.86 ± 2.76 kg/m^2^. Percentage of smoking was 74.3% (589/793, including historical and present of smoking) while percentage of drinking 63.8% (506/793, including past and present drinking). The racial composition of the population was mostly Chinese Han (85.8%). The reported percentage of insomnia at 500 m was 14.5% (115 out of 793).

Of all participants, 73.3% experienced HAH upon acute exposure at 3700 m. Physiological and psychological parameters with statistical differences from 500 m to 3700 m were summarized in Table 1, including systolic blood pressure (SBP), diastolic blood pressure (DBP), mean arterial blood pressure (MAP), HR, pulse pressure(ΔBP) SaO_2_, SAS, and ESS (all of the *p*-value <0.001)*.*

**Table 1:** Physiological and psychological parameters at 500 m and 3700 m

	**500 m**	**3700 m**	**Change**	***p *value**
SBP	115.23±10.51	119.03±12.16	10.65±8.32	<0.001
DBP	72.71±9.29	78.90±10.20	10.30±	<0.001
ΔBP	43.52±7.73	40.13±8.06	8.33±7.59	<0.001
MAP	86.82±9.06	92.27±10.20	9.81±8.02	<0.001
SaO_2_	98.09±1.03	88.77±3.16	9.34±3.21	<0.001
HR	65.92±10.40	84.81±12.55	20.06±11.80	<0.001
SAS	21(3)	24(6)	7(10)	<0.001
ESS	11(4)	12(5)	2(4)	<0.001

SBP, DBP, MAP and ΔBP: mmHg; SaO_2_:%; HR: beat per min (bpm); SAS and ESS: score; bracket: inter-quartile range. Comparison of SBP, DBP, MAP, ΔBP, SaO_2_ and HR were employed by paired-sample T test between 500 m and 3700 m altitude. SAS and ESS were compared by Mann–Whitney U-test.

Percentages of primary headache history (*p* <0.001) and insomnia (*p* <0.001) were significantly higher in HAH+ group than HAH- group (Table 2). Mean age was higher in HAH+ group than HAH- group (*p* =0.011). HAH+ subjects with a higher HR (*p* =0.002), SAS (*p* <0.001) and ESS (*p* <0.001) but a lower SaO_2_ (*p* =0.001) than HAH- subjects. PLI was also significantly different between HAH+ and HAH- groups (*p* =0.044) (Table 2). The abovementioned factors were selected into preliminary screening of the logistic regression model for each single variable.

**Table 2:** Risk factors in various degrees of HAH

**Headache Cases(n)**	**HAH-(212)**	**HAH+(581)**	**HAH intensities**			***p *(HAH+ *vs*. HAH-)**
			**1(487)**	**2(88)**	**3(6)**	
Demographic factors						
Age	22.49±3.42	23.23±4.18	23.17±4.12	23.51±4.20	24.64±3.67	0.011
BMI	21.71±2.52	21.92±2.84	21.91±2.91	21.86±2.50	23.34±2.47	0.351
Smoking						0.740
0	43(20.3)	161(27.7)	139(28.5)	22(25.0)	0(0)	
1	133(62.7)	287(49.4)	240(49.3)	43(48.9)	4(66.7)	
2	36(17.0)	133(22.9)	108(22.2)	23(26.1)	2(33.3)	
Alcohol consumption						0.748
0	72(34.0)	215(37.0)	182(37.4)	32(36.4)	1(16.7)	
1	16(7.5)	26(4.5)	23(4.7)	3(3.4)	0(0)	
2	124(58.5)	340(58.5)	282(57.9)	53(60.2)	5(83.3)	
History						
Primary headache(yes)	12(5.7)	124(21.3)	86(17.7)	34(38.6)	4(66.7)	<0.001
High altitude exposure (yes)	64(30.2)	156(26.9)	127(26.1)	24(27.3)	5(83.3)	0.353
Athletic training(yes)	23(10.8)	72(12.4)	56(11.5)	16(18.2)	0(0)	0.554
PLI						0.044
1	45(21.2)	156(26.8)	132(27.1)	23(26.1)	1(16.7)	
2	103(48.6)	284(48.9)	232(47.6)	49(55.7)	3(50.0)	
3	64(30.2)	141(24.3)	123(25.3)	16(18.2)	2(33.3)	
At 3700 m						
Psychological scale						
SAS	22(4)	24(6)	24(5)	27(10)	28.5(8.75)	<0.001
Physiological factors						
SBP	118.50±11.04	119.23±12.55	119.04±12.53	120.47±12.88	115.83±8.47	0.458
DBP	78.57±10.03	79.02±10.26	78.92±10.22	79.72±10.60	76.33±8.96	0.582
ΔBPBP	39.93±7.13	40.21±8.38	40.12±8.29	40.75±9.06	39.50±5.17	0.672
MAP	91.88±9.82	92.42±10.35	92.30±10.33	93.30±10.58	89.50±8.46	0.508
SaO_2_	89.41±2.74	88.54±3.27	88.67±3.24	87.82±3.38	87.83±3.06	0.001
HR	82.49±11.69	85.66±12.76	85.13±12.39	88.22±13.76	90.50±22.23	0.002
Sleep						
Insomnia(yes)	95(44.8)	393(67.6)	312(64.1)	76(86.4)	5(83.3)	<0.001
ESS	11(4.5)	12(5)	12(5)	14(5)	12(7.25)	<0.001

Independent T tests were used to compare two means of SBP, DBP,ΔBP, MAP, SaO_2_, and HR between HAH+ and HAH- groups at 3700 m. The Mann–Whitney U-test was applied to evaluate differences between ordinal or non-normally distributed data (PLI, smoking, alcohol consumption, SAS and ESS).

People with insomnia had higher rate of HAH than those without insomnia (80.5% *vs.* 60.0%, *p* <0.001). Individuals with a history of primary headache reported more HAH than those without a primary history (91.2% *vs.* 69.3%, *p* <0.001).

The severity of HAH was related to age (r =0.073, *p* =0.039), HR (r =0.122, *p* =0.001), SaO_2_ (r =-0.144, *p* <0.001), SAS (r =0.360, *p* <0.001) and ESS (r =0.186, *p* <0.001).

Each single variable was analyzed by logistic regression in the preliminary screening. Age, primary headache history, SAS, SaO_2_, HR, insomnia, ESS and PLI were included in the next analysis for independent risk factors (all of the *p*-value <0.05). However, the variables below were not used in the next adjusted logistic regression models due to the *p* >0.05, including BMI, smoking history, alcohol consumption history, previous high altitude exposure, athletic training, SBP, DBP, MAP and ΔBP (Table 3).

**Table 3:** Logistic regression analysis for each single variable and adjusted independent risk factors

**Variable**	**β-coefficient**	**Odds ratio**	**(95% CI)**^**a**^	***p *value**
Demographic factors				
Age	0.05	1.05	1.01–1.10	0.020
BMI	0.03	1.03	0.97–1.09	0.352
Smoking	0.03	0.97	0.77–1.22	0.790
Alcohol consumption	–0.04	0.96	0.82–1.14	0.680
History				
Primary headache(yes)	1.51	4.52	2.44–8.37	<0.001
High altitude exposure (yes)	–1.64	0.85	0.60–1.20	0.353
Athletic training(yes)	0.15	1.16	0.71–1.91	0.554
Psychological scale				
SAS	0.20	1.22	1.16–1.29	<0.001
Physiological factors				
SBP	0.01	1.01	0.99–1.02	0.457
DBP	0	1.00	0.99–1.02	0.581
ΔBPBP	0	1.00	0.98–1.02	0.671
MAP	0.01	1.00	0.99–1.02	0.507
SaO_2_	–0.09	0.91	0.86–0.96	0.001
HR	0.02	1.02	1.01–1.04	0.002
Sleep				
Insomnia(yes)	0.97	2.64	1.92–3.65	<0.001
ESS	0.09	1.09	1.05–1.14	<0.001
PLI	–0.23	0.80	0.64–1.00	0.045
Adjusted independent risk factors				
Primary headache history (yes)	1.20	3.30	1.73–6.30	<0.001
Insomnia(yes)	0.65	1.91	1.35–2.70	<0.001
SaO_2_	–0.06	0.94	0.89–1.00	0.035
HR	0.020	1.020	1.00–1.03	0.009
SAS	0.16	1.18	1.11–1.25	<0.001

a: 95% CI: 95% confidence intervals. A binary logistic regression model was used to identify the risk factors for HAH.

Analysis by logistic regression revealed that the following factors were the independent ones for HAH including primary headache history (OR: 3.30; 95% CI: 1.73–6.30; *p* <0.001), insomnia (OR: 1.91; 95% CI: 1.35–2.70; *p* <0.001), low SaO_2_ (OR: 0.94; 95% CI: 0.89–1.00; *p* =0.035), high HR (OR: 1.02; 95% CI: 1.01–1.03; *p* =0.009) and SAS (OR: 1.18; 95% CI: 1.11–1.25; *p* <0.001). They were all strongly associated with HAH (Table 3).

## Discussion

The stress of high-altitude hypobaric hypoxia may cause the pathogenesis of HAH. As demonstrated herein, occurrence of HAH was influenced by several factors.

### Relationship between HAH and anxiety after acute high altitude exposure

High-altitude hypobaric hypoxia is a challenge to the psychological state and physiological condition of human beings. The association of cephalalgia with anxiety disorders has also been indicated by several studies [16]. However, the relationship between anxiety or SAS and HAH is not confirmed. In our study, SAS would reflect the extent of anxiety substantially in recent time at high altitude though it was performed within 24 hours after arrival at 3700 m. The SAS score which reflected level of anxiety state was higher in HAH+ group than that in HAH- groups. We observed that the high SAS score was closely related to HAH (the median SAS score was 24 in HAH+ and 22 in HAH- subjects), even though few subjects (5 cases) were confirmed to have an anxiety disorder (SAS score>40). Thus, our findings together with a previous one [17] suggest that anxiety and psychosocial stress could be used as predictors of headache.

### Relationship between sleep and HAH at high altitude

There is a bidirectional relationship between sleep disturbance and cephalalgia at low altitude [18]. A previous study revealed that transient recurrent situational insomnia was associated with headache [11]. On the other hand, another research indicated that individuals with headache were more likely to report insomnia[12]. Insomnia increased the rate of HAH in our study, which is consistent with the study that people with insomnia have an increased risk of recurrent headache at low altitude [12].

Lethargy is a common subjective complaint upon high-altitude exposure. ESS is not only a subjective measure of daytime sleepiness or sleep propensity but also an evaluation for objective sleepiness [19,20]. Although it was significantly different between HAH+ and HAH- groups in our study, ESS score was not a risk factor for HAH after adjusting for other factors, such as anxiety.

Although we could not exclude that at high altitude HAH caused insomnia at night, it is necessary to relieve the symptoms of both HAH and insomnia. Before and after arrival at high-altitude maintaining adequate sleep and preventing HAH will be helpful.

### History affecting the HAH development

History of primary headache is a risk factor of HAH in our study, which is in agreement with that migraine increased both the frequency and severity of HAH [21], but in contrast to a study that migraine history did not affect HAH [22]. The inconsistencies among these investigations may be due to the variations of sample sizes, altitudes and ascending speeds. Thus, research on headache provides a possible theoretical and methodological basis for studying HAH.

We found that histories of high-altitude exposure and athletic training were not risk factors for HAH, similar to the report [10] that history of pre-exposure >2000 m was not different between HAH+ and HAH- groups. Neither history of high-altitude exposure nor athletic training is closely associated with HAH, possibly due to the athletic training intensity and period without conformity.

### HAH and cardiovascular system alternations

HR increased rapidly upon high-altitude (3700 m) exposure and increased HAH in our study, similar to another research on HAH risk factors in mountaineers [10]. In this study, SBP, DBP, MAP and ΔBP were not different between HAH+ and HAH- groups. While augmented BP may cause headache, a slight rise in BP does not trigger a strong headache.

We also found that SaO_2_, an indicator of oxygen supply to tissues and organs, was negatively correlated to HAH due to desaturation of oxygen and hypoxia, a finding consistent with a previous study [10]. Thus, SaO_2_ is a protective determinant of HAH, which can be improved by oxygen inhalation to prevent HAH and AMS.

### Demographic factors and HAH

Age and BMI did not contribute to HAH in our study, a similar outcome to that of other studies [10], possibly because all subjects were healthy young men (23.03±4.01 years old). However, age was related to the severity of HAH. Subjects in HAH+ group were older than those in HAH- one, which is contrary to another study [9]. BMI (21.86±2.76 kg/m^2^), which was concentrated in the normal healthy range, was not closely related to HAH.

Our study did not reveal any associations between HAH and cigarette or alcohol consumption. However, smokers with high headache prevalence than non-smokers at low altitude [23]. It has been indicated that alcohol consumption reduces migraine [23]. Therefore, we surmise that the response to acute altitude exposure through vasoconstriction, change of cerebrovascular reactivity and other physiological compensatory reactions disguised the effect of smoking and alcohol consumption on headache, and that the smoking and drinking indexes (or cumulative amounts of smoking and drinking) did not affect HAH.

PLI was not an independent risk factor associated with HAH, though it was significantly different between HAH+ and HAH- groups. However, studies have found that low physical activity is associated with the higher prevalence of migraines [24]. Such contradiction may be attributed to our non-precise quantitative physical labor intensity.

In addition, the existing study on the risk factors of HAH was performed over a longer time period with a wider height spectrum (from 2200 to 3817 m) [10], where HAH may not be typical. Our study was performed within 24 hours after arrival at 3700 m by plane within two hours, giving them “acute altitude exposure”. Thus, the homogeneity of our study is more representative. Furthermore, we also included psychological risk factors in our study.

### Limitations

As Lawley et al. suggested that the researchers should not label all headache as HAH just because their presence was reported above 500 m [5], we screened headache that occurred at high altitude. In this study, headache basically satisfied the diagnostic criteria of the International Classification of Headache Disorders, although headache characteristics were not detailedly recorded in CRFs, which should be improved in the future studies. Although the descent was not performed immediately after the onset of the headache, cephalalgia almost resolved after a five days’ rest at 3700 m [25.6%, 153 out of 597 (mild: 143, moderate: 9, severe: 1)]. In addition, there are many other potential risk factors that have not been considered in this study (such as family factors).

## Conclusions

Insomnia is a risk factor for HAH, but high ESS is not, which reveals the relationship between sleep and HAH. History of primary headache is also a risk factor for HAH. The physiological factors, low SaO_2_ and high HR are related to a high probability of HAH. Additionally, a high SAS score remains hazardous for HAH. Reducing the risk factors may be approaches to decrease HAH and protect individuals who ascend to high altitudes acutely. However, demographic factors, such as age, are not independent risk factor. Identification of the risk factors for HAH provides information for the prevention and treatment of HAH.

## Abbreviations

AMS: Acute mountain sickness; SBP: Systolic blood pressure; DBP: Diastolic blood pressure; BP: Blood pressure; MAP: Mean arterial blood pressure; ΔBP: Pulse pressure; SaO2: Arterial oxygen saturation; HR: Heart rate; HAH+: With high-altitude headache; HAH-: Without high-altitude headache; Per: Percentage; SAS: Self-rating anxiety scale; ESS: Epworth sleepiness scale; CI: confidence intervals.

## Competing interest

The authors declare that there is no conflict of interest.

## Authors’ contributions

SZB and LH designed this research. SZB performed the statistical analysis and drafted the manuscript. JHZ, XBG and LH critically reviewed and revised this manuscript for important intellectual inputs. SZB, ML, JY, XL, JQD, and GZC carried out the filling of CRFs and the measurements of BP, HR and SaO_2_. All authors read and approved the final manuscript.

## Supplementary Material

Additional file 1 Self-Rating Anxiety Scale. Click here for file

Additional file 2 Epworth Sleepiness Scale. Click here for file
