# Supplementary material for: Risk factors for high-altitude headache upon acute high-altitude exposure at 3700 m in young Chinese men: a cohort study
Source: J Headache Pain. 2013 Apr 11;14(1):35. doi: 10.1186/1129-2377-14-35 (PMC3630053; doi:10.1186/1129-2377-14-35)
Supplement: Additional file 2 — Epworth Sleepiness Scale. [file 1129-2377-14-35-S2.doc]

*Additional file 2*

Epworth Sleepiness Scale

How likely are you to dozen off or fall asleep in the following situations in contrast to feeling just tired? This refers to your usual way of life in recent times.

Even if you haven’t done some of these finding recently try to work out how they would have affected you.

Use the following scale to choose the most appropriate number for each situation:

0 = would never doze

1 = slight chance of dozing

2 = moderate chance of dozing

3 = high chance of dozing

It is important that you answer each question as best as you can.

| Situation | Chance of dozing (0-3) |
| --- | --- |
| 1.Sitting and reading |  |
| 2.Watching TV |  |
| 3.Sitting ,inactive in a public place(eg, a theater or meeting) |  |
| 4.As a passenger in a car for an hour without break |  |
| 5.Lying down to rest in the afternoon when circumstances permit |  |
| 6.Sitting and talking to someone |  |
| 7.Sitting quietly after a lunch without alcohol |  |
| 8.In a car, while stopped for a few minutes in traffic |  |
| *Thank you for your cooperation.* | |

They were translated into Chinese when used in the trial.
